# Supplementary material for: Aharonov–Bohm oscillations in Dirac semimetal Cd3As2 nanowires
Source: Nat Commun. 2016 Feb 23;7:10769. doi: 10.1038/ncomms10769 (PMC4766419; doi:10.1038/ncomms10769)
Supplement: Supplementary Information — Supplementary Figures 1-9. [file ncomms10769-s1.pdf]

Supplementary Figures

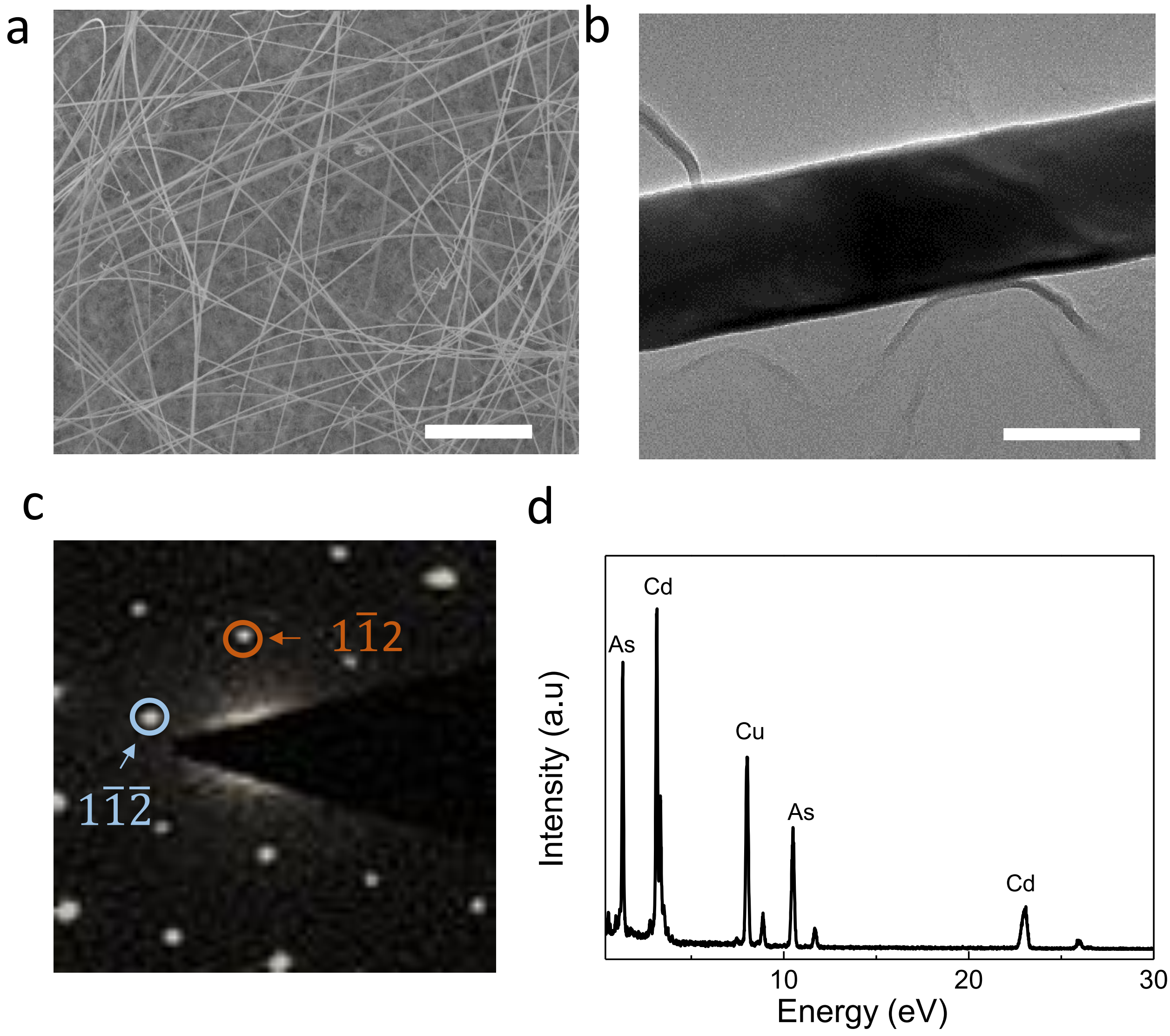

Supplementary Figure 1 | Characterization of Cd_3As_2 nanowires. (**a**) SEM image (Scale bar: 20 μm) and (**b**) TEM image (Scale bar: 100 nm) of Cd_3As_2 nanowires. (**c**) Typical electron diffraction pattern and (**d**) the energy-dispersive X-ray spectroscopy (EDS) results of Cd_3As_2 nanowires. The as-grown Cd_3As_2 nanowires manifest great flexibility. The nanowire diameter is in the range from tens of nanometers to several hundreds of nanometers. The selected electron diffraction pattern in (**c**) indicates the growth direction is along the [112] crystalline axis. The EDS results in (**d**) show the right stoichiometry of the Cd_3As_2 compound.

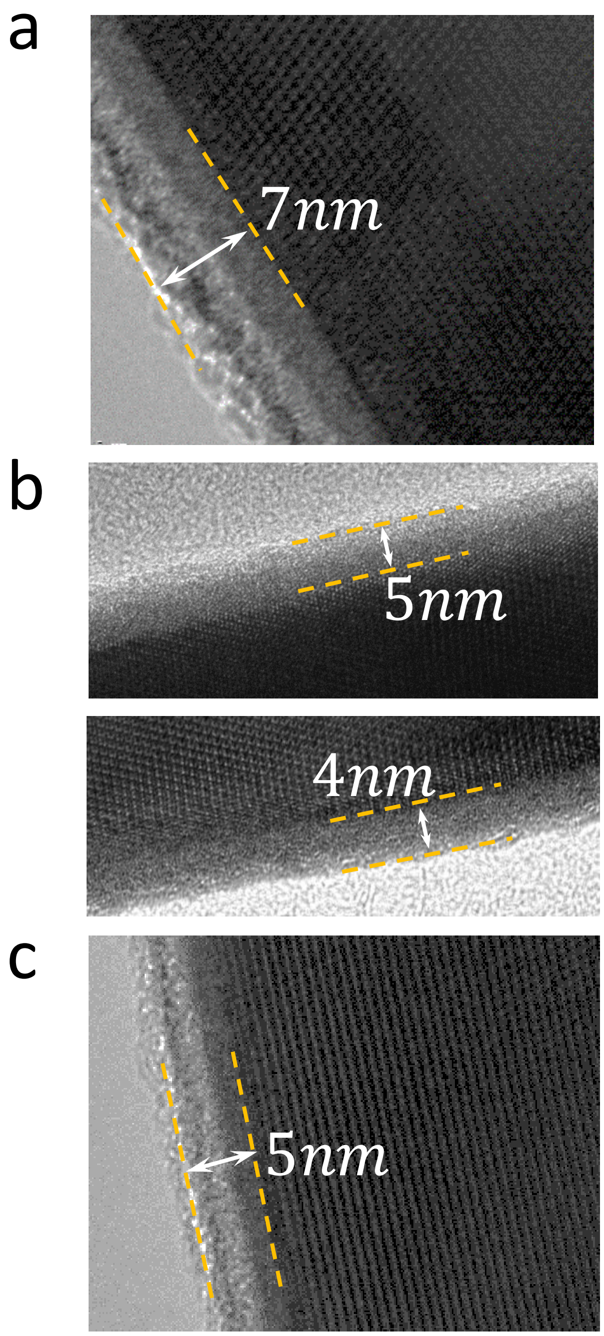

Supplementary Figure 2 | Amorphous layer on the nanowire surface. High resolution TEM images of several typical Cd_3As_2 nanowires. Amorphous layer on Cd_3As_2 nanowires is generally observed in the TEM images, which may be due to the natural oxidation of the surface after exposed in the air.

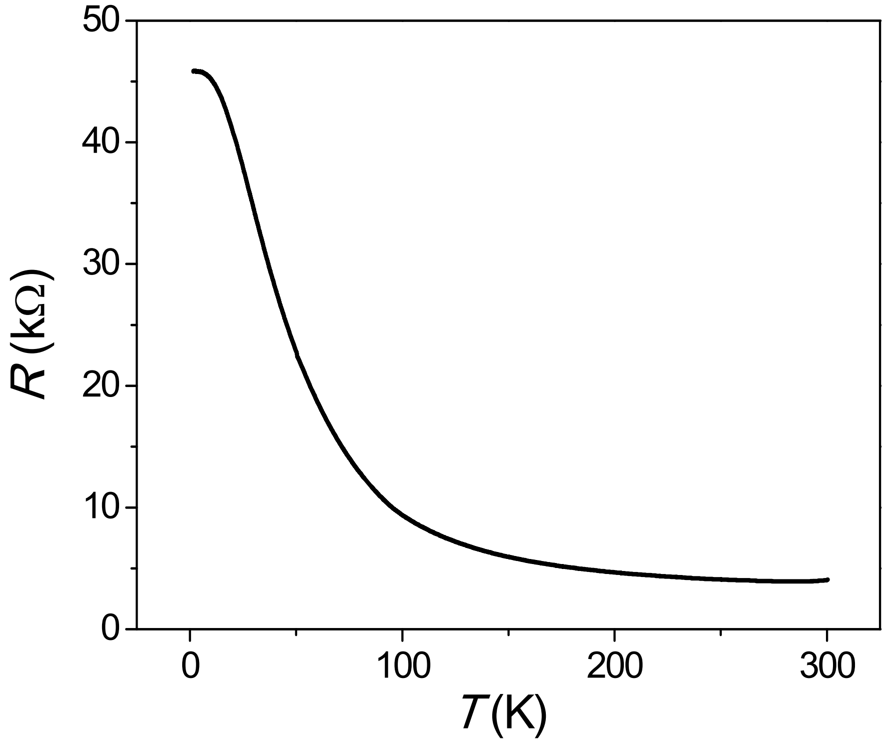

Supplementary Figure 3 | Resistance as a function of temperature. The semiconducting-like *R*-*T* behavior indicates that the Fermi level is close to the Dirac point due the low carrier density of the nanowire. The thermal energy can activate the carriers to enhance the conductance, resulting in the resistance decreases with increasing temperature.

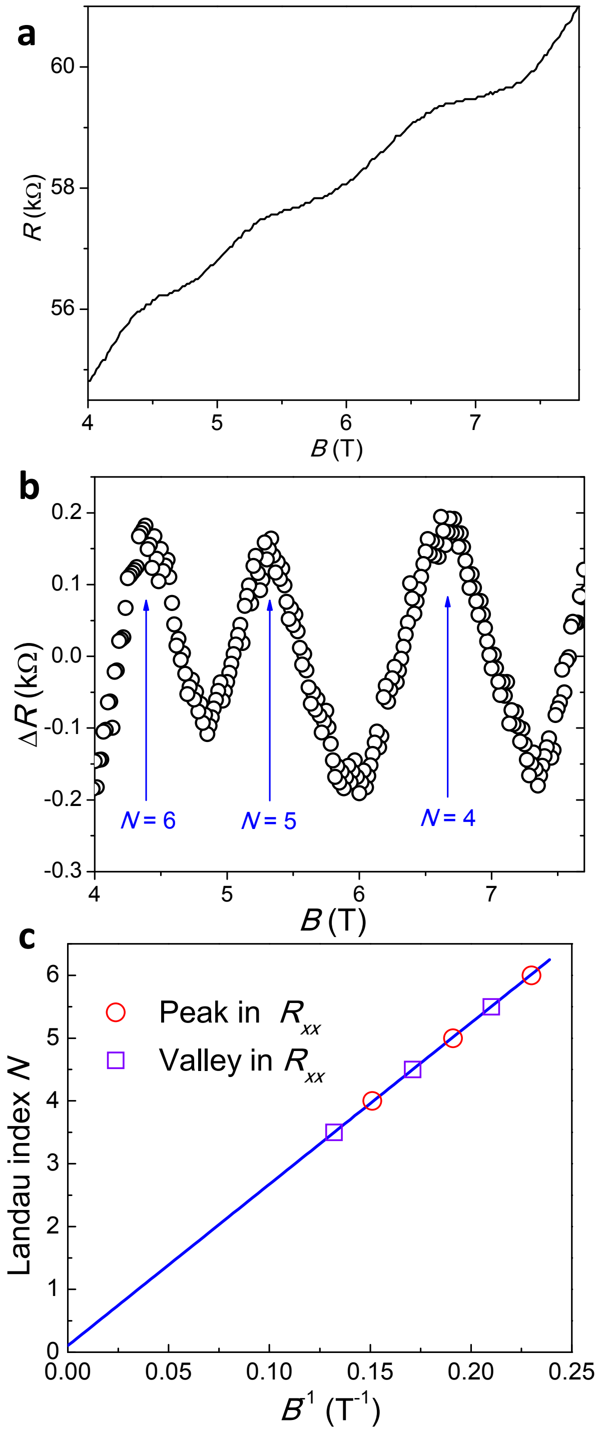

Supplementary Figure 4 | Shubnikov-de Haas oscillations. (**a**) The SdH oscillations in the plot of resistance R *vs.* magnetic field B. (**b**) Extracted oscillation part ΔR as a function of magnetic field B. (**c**) Landau index plot by assigning integers to the maxima in ΔR, yielding a intercept of 0.1 by the linear extrapolation.

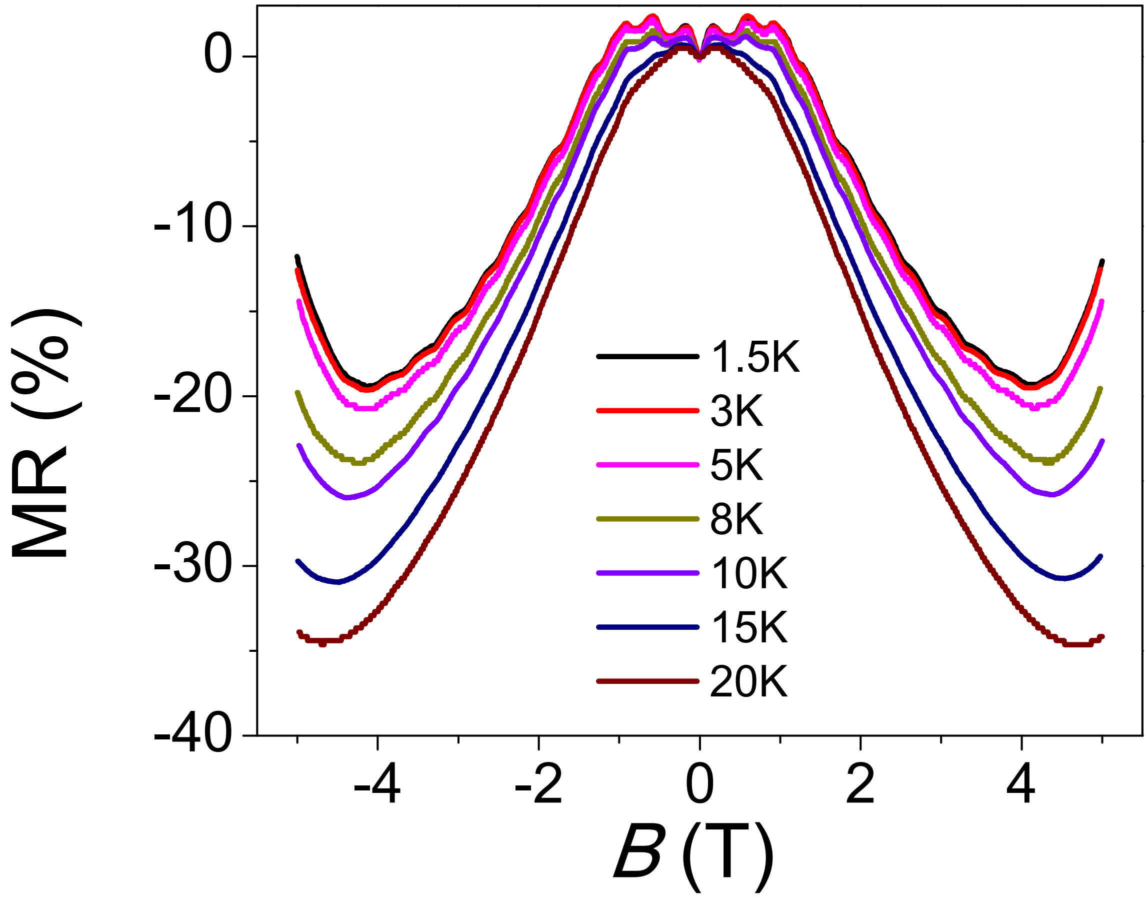

Supplementary Figure 5 | Negative magnetoresistance. Magnetoresistance, defined as MR=[$R(B)/R(0)$-1]×100%, of Cd_3As_2 nanowires at variable temperatures in $B//E$ configuration shows negative values, giving signature of the chiral anomaly effect.

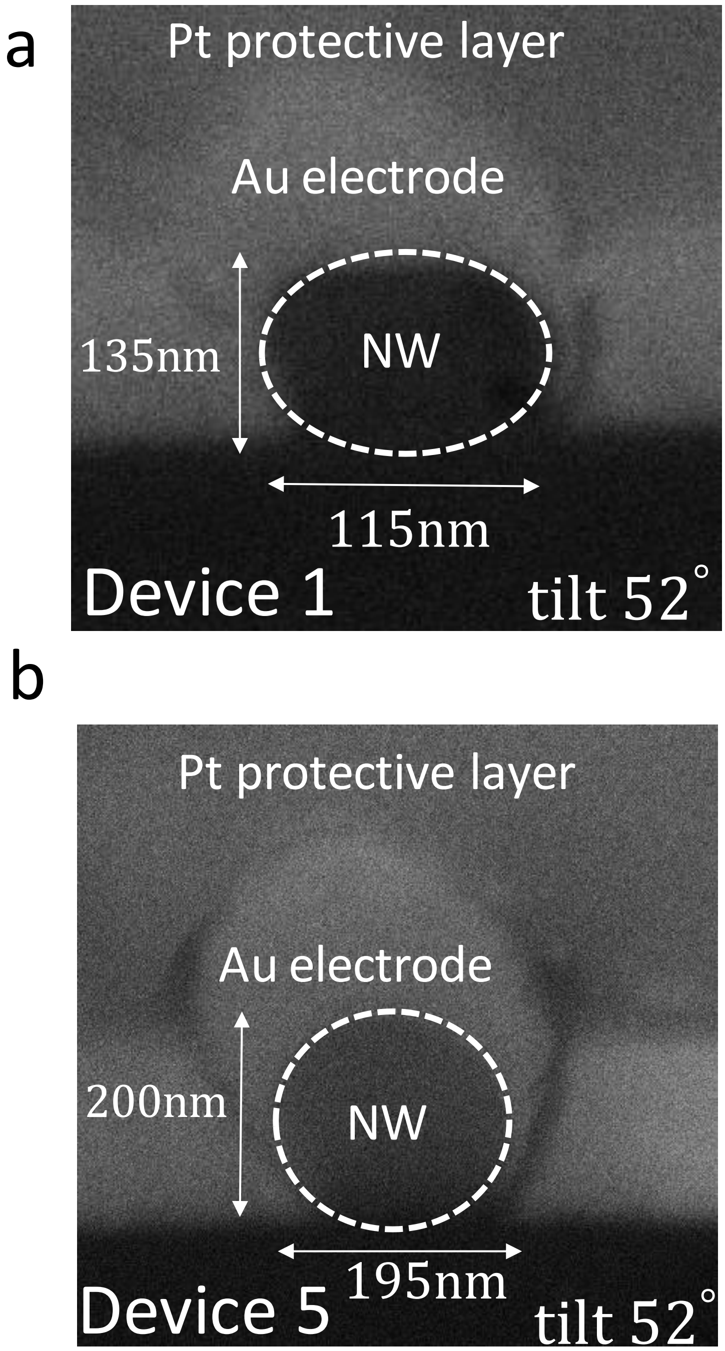

Supplementary Figure 6 | Cross-section of Devices 1 and 5. SEM images at tilt angle 52° of nanowire cross-section of (**a**) Device 1 and (**b**) Device 5. To acquire the cross-sectional area of the nanowire devices, a thin Pt film was first deposited on the nanowire as a protective layer by electron beam induced deposition method in a dual beam system (Dual-Beam 235-FIB system, FEI Company). Then, the nanowires were cut by focused ion beam.

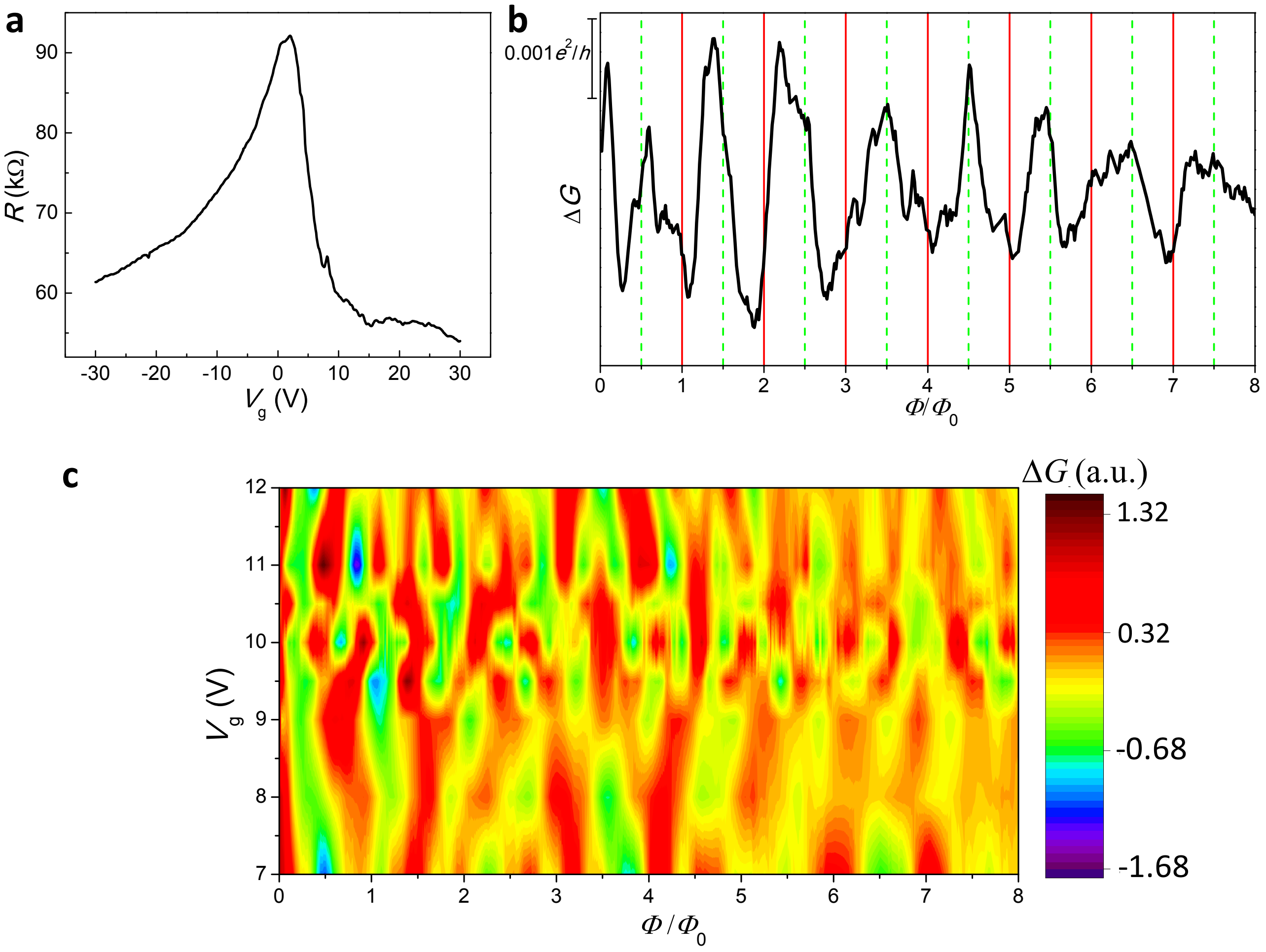

Supplementary Figure 7 | Gate modulated transport properties of a nanowire with radius of ~37 nm (Device 3). (**a**) Transfer curve at 1.5 K. (**b**) Oscillation part ΔG as a function of magnetic flux in unit of $\Phi_0 = h/e$ at $V_g = 10.5$ V. (**c**) Mapping of gate-dependent conductance oscillations from $V_g =$ 7 to 12 V with a step of 0.5 V. For comparation, the ΔG was normalized.

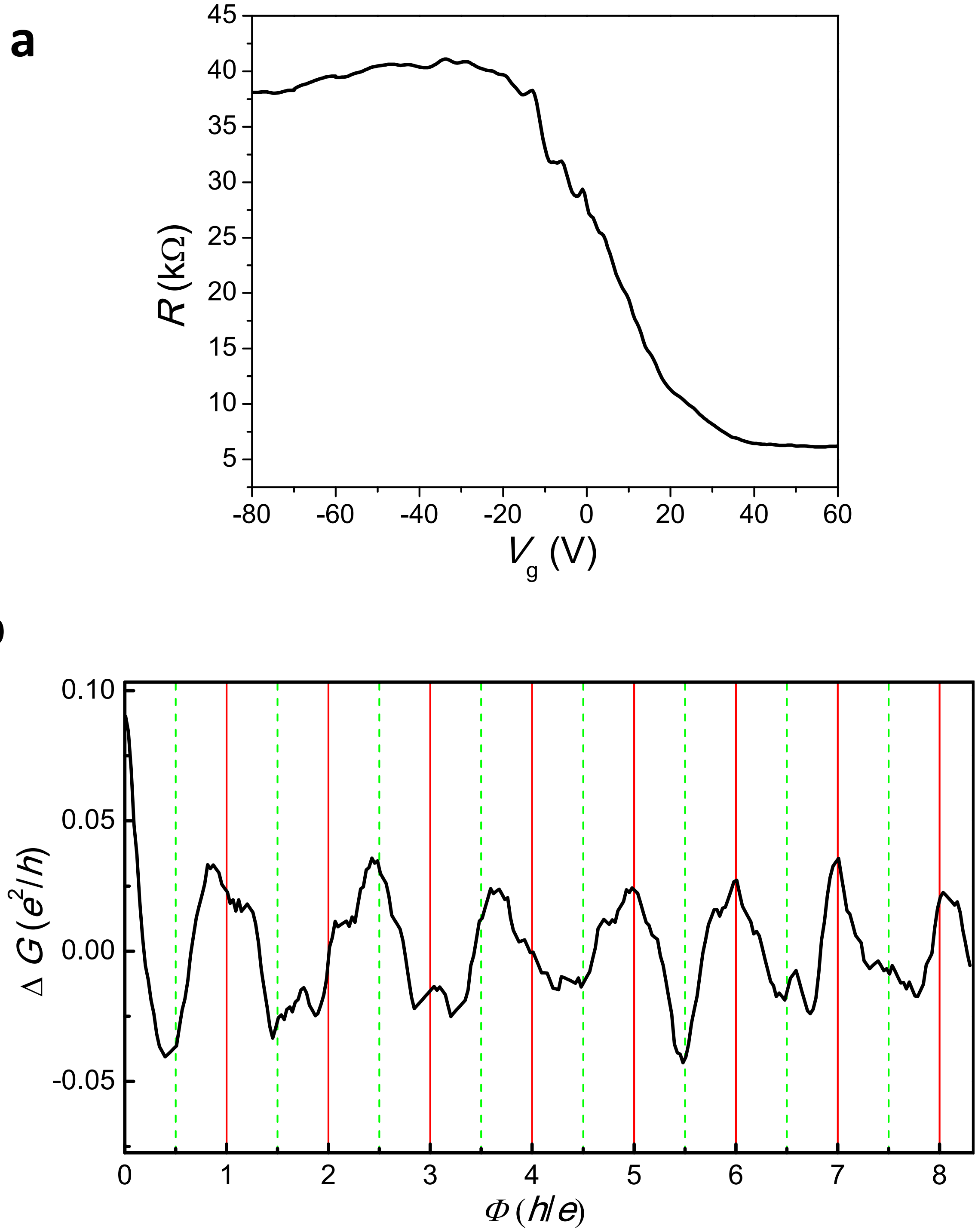

Supplementary Figure 8 | Gate modulated transport properties of a nanowire with diameter of ~90 nm (Device 4). (**a**) Resistance as a function of applied gate-voltage V_g at 1.5 K. (**b**) A-B oscillations as a function of Φ at V_g = 60 V after subtracting the background.

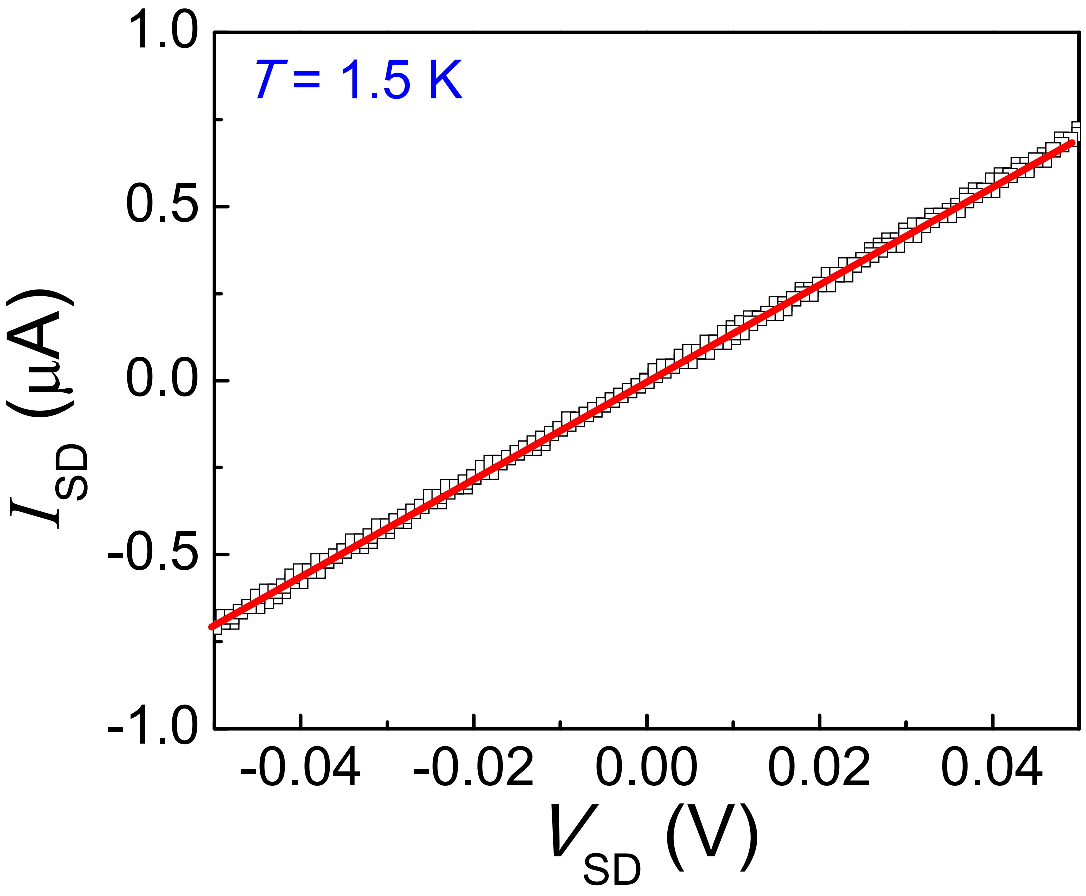

Supplementary Figure 9 | Current-voltage curve of Device 5 at 1.5 K. The linear source-drain current I_{SD} *vs.* source-drain voltage V_{SD} curve indicates the Ohmic contacts between Au electrodes and Cd_3As_2 nanowires.
